# Cimifugin Suppresses NF-κB Signaling to Prevent Osteoclastogenesis and Periprosthetic Osteolysis

**DOI:** 10.3389/fphar.2021.724256

**Published:** 2021-09-29

**Authors:** Juan Duan, Xuantao Hu, Tao Li, Gen Wu, Pengcheng Dou, Zhengxiao Ouyang

**Affiliations:** ^1^ Department of Geriatric Internal Medicine, The Second Xiangya Hospital, Central South University, Changsha, China; ^2^ Deparment of Orthopedics, The Second Xiangya Hospital, Central South University, Changsha, China

**Keywords:** osteoclast, NF-κB, p38, MAPK, aseptic prosthetic loosening, periprosthetic osteolysis, cimifugin

## Abstract

**Background:** Aseptic loosening of prosthesis (ALP) is one of the most common long-term complications of knee and hip arthroplasty. Wear particle-induced osteoclastogenesis and subsequent periprosthetic osteolysis account for the morbidity of ALP. Here, we investigate the potential of cimifugin (CIM), a natural extract from Cimicifuga racemosa and *Saposhnikovia divaricata*, as a bone-protective drug in the treatment of ALP.

**Method:** First, we performed cell viability and osteoclast formation assays to assess the effect of noncytotoxic CIM on osteoclast differentiation *in vitro*. Bone slice resorption and F-actin ring immunofluorescence assays were adopted to assess the effects of CIM on bone-resorption function. Then, quantitative real-time polymerase chain reaction (qRT–PCR) analysis was performed to further assess the repressive effects of CIM on osteoclastogenesis at the gene expression level. To elucidate the mechanisms underlying the above findings, Western blot and luciferase reporter gene assays were used to assess the regulatory effects of CIM on the NF-κB and MAPK signaling pathways. Moreover, a Ti particle-induced murine calvarial osteolysis model and subsequent histomorphometric analysis via micro-CT and immunohistochemical staining were used to elucidate the effect of CIM on periprosthetic osteolysis *in vivo*.

**Result:** CIM dose-dependently inhibited both bone marrow-derived macrophage (BMM)- and RAW264.7 cell-derived osteoclastogenesis and bone resorption pit formation *in vitro*, which was further supported by the reduced expression of F-actin and osteoclast-specific genes. According to the Western blot analysis, inhibition of IκBα phosphorylation in the NF-κB signaling pathway, not the phosphorylation of MAPKs, was responsible for the suppressive effect of CIM on osteoclastogenesis. Animal experiments demonstrated that CIM alleviated Ti particle-induced bone erosion and osteoclast accumulation in murine calvaria.

**Conclusion:** The current study suggested for the first time that CIM can inhibit RANKL-induced osetoclastogenesis by suppressing the NF-κB signaling pathway *in vitro* and prevent periprosthetic osteolysis *in vivo*. These findings suggest the potential of CIM as a therapeutic in ALP.

## Introduction

With the development of prosthesis design and minimally invasive procedures, arthroplasty has become one of the most reliable surgical procedures to treat stubborn diseases, such as severe osteoarthritis, developmental hip dysplasia, femoral neck fractures, rheumatoid arthropathy, and ankylosing spondylitis. In recent decades, the number of people receiving arthroplasty surgery worldwide has continuously increased by hundreds of thousands per year. Research suggests that approximately 5% of patients develop aseptic loosening of prosthesis (ALP) after undergoing arthroplasty and require complicated revision surgeries (Goodman and Gallo, 2019). To avoid this, substantial efforts have been made to find therapies for ALP.

The pathophysiology of ALP has been mainly attributed to the following two aspects: the “stress shielding” caused by highly rigid prostheses and the wear particles produced by the friction between implants and host bone (Panegrossi et al., 2014; Visgauss et al., 2020). Generally, nanoparticles shed from Ti-, Co- and Cr-based metals, polyethylene, bone cement and ceramic implants trigger an inflammatory response in periprosthetic tissue (Zhang et al., 2020; Prock-Gibbs et al., 2021). Cell lineages, including macrophages, osteoblasts, osteoclasts, and mesenchymal stem cells, participate in the mechanistic network and produce a surge of proinflammatory factors, including reactive oxygen species, chemokines, TNF-α, IL-1β, and IL-6 (Sun et al., 2019). Notably, disruption of osteoblast metabolism causes an imbalance between the receptor activator of nuclear factor κ B ligand (RANKL) and osteoprotegerin (OPG) (Kim et al., 2020). Excessive RANKL triggers the formation of osteoclasts by binding to the receptor activator of nuclear factor κ B (RANK) located on the surface of the cell membrane and consequently produces an initial signal to recruit TNF receptor-associated factor 6 (TRAF6) (Walsh et al., 2015). Activated TRAF6 promotes the nuclear factor κB (NF-κB) and mitogen-activated protein kinase (MAPK) signaling cascades, thereby leading to the upregulation of osteoclast marker genes, including Nfatc1, Calcr, and Ctsk. As a result, bone-resorbing effect-mediated osteoclasts are enhanced, and periprosthetic osteolysis occurs (Walsh et al., 2015). Therefore, the wear debris-induced inflammatory response and pathological formation of osteoclasts in periprosthetic tissues play pivotal roles in the occurrence of ALP.

To prevent ALP, the design of the total hip arthroplasty prosthesis has been improved over several generations to guarantee proper stress distribution and reduce the abrasion between interfaces by adopting biochemically conforming shapes and materials of higher biocompatibility. However, for patients with risk factors and for those who have already developed early-stage ALP, the effects of existing treatments are less than satisfactory (Hu et al., 2020). Given this, we investigated a natural compound called cimifugin (CIM), one of the main components of *Saposhnikovia divaricata* and Cimicifuga racemosa extract (He et al., 2000; Wang et al., 2017). CIM is commonly reported to be a bioactive chromone that exists independently or in multiple Chinese medicine formulae and exerts antinociceptive, anti-inflammatory and lipid metabolism regulatory effects on a wide range of diseases, including skin and respiratory allergies, inflammatory arthropathies, and cerebral ischemia, by inhibiting the production of inflammatory factors and NF-κB/MAPK signaling (Wu et al., 2016; Wang et al., 2017; Han et al., 2019; Jia et al., 2019; Yao et al., 2019; Liu et al., 2020). However, regarding ALP treatment, the bioactivity of CIM in osteoclastogenesis and subsequent periprosthetic osteolysis has not been investigated. Additionally, the molecular mechanisms involving osteoclasts and their precursors remain elusive.

To this end, we designed *in vitro* and *in vivo* experiments in the current study and attempted to 1) investigate the effect of CIM on osteoclast differentiation, 2) detect the therapeutic effect of CIM on Ti particle-induced periprosthetic osteolysis and 3) elucidate the underlying molecular mechanism. The current findings suggest an extension of the natural compound spectrum for ALP prevention or conservative treatment.

## Materials and Methods

### Cell Culture and Main Reagents

The cell lineages used in the present study included osteoclast precursor RAW 264.7 cells and murine primary bone marrow monocytes (BMMs). The RAW264.7 cells were obtained from the Orthopedic Laboratory of the Second Xiangya Hospital and cultured in standard alpha-modified minimal essential medium (α-MEM) (Gibco-BRL; Beijing, China) supplemented with 10% fetal bovine serum (FBS) (Gibco-BRL; Scotland, United Kingdom) and 1% penicillin/streptomycin (termed complete α-MEM, c-α-MEM). The cells were culture in an incubator under standard conditions, including a constant temperature of 37°C and a 5% CO_2_ humidified atmosphere (Zhang et al., 2018). BMMs were isolated as described in a previous study (Ouyang et al., 2014). In brief, bone marrow was harvested from the bone shafts of male, four-to six-week-old C57BL/6 mice and cultured in c-α-MEM supplemented with 10 ng/m l macrophage colony-stimulating (M-CSF) (R and D Systems; Minneapolis, MN, United States) for 24 h. Then, nonadherent cells were removed, and the adherent cells (primary BMMs) were continuously incubated in fresh c-α-MEM with M-CSF for 72–96 h until reaching confluence. The medium and incubation conditions used in the present study remained unchanged unless stated otherwise.

CIM (C_16_H_18_O_6_, 306.310, purity ≥99%) was purchased from Yuanye Biology Ltd. (Shanghai, China) and dissolved in α-MEM at 80 mM as a storage solution at 4°C in the dark. RANKL was acquired from R and D Systems (Minneapolis, MN, United States). Cell counting kit 8 (CCK-8) was purchased from Dojindo Molecular Technology (Shanghai, China). The tartrate-resistant acid phosphatase (TRAP) staining kit was purchased from Sigma Aldrich (St Louis, MO, United States). Primary and secondary antibodies for Western blotting were purchased from Cell Signaling Technology (Cambridge, MA, United States). A Qiagen RNeasy Mini kit (Qiagen, Valencia, CA, United States) and reverse transcriptase (TaKaRa Biotechnology, Otsu, Japan) SYBR Premix Ex Taq kit (TaKaRa, Biotechnology, Otsu, Japan) were used for qPCR.

### Cell Viability

To assess the cytotoxicity of CIM, RAW264.7 or BMM cells were seeded at 3 × 10^3^/well in triplicate in 96-well plates and incubated overnight to allow adhesion. Then, CIM was added to the medium at a gradient of concentrations (0, 10, 20, 40, 80, 160, 320, 640, 1,280, 2,560 μM) for 48 or 96 h. The medium was replaced every 48 h. Then, the medium in each well was replaced with 100 µL of fresh medium supplemented with a 10% CCK-8 buffer solution, and the 96-well plates were incubated under the same conditions for 1 h. The absorbance was measured at a wavelength of 450 nm using an ELX800 absorbance microplate reader (Bio-Tek, United States), and phosphate-buffered saline (PBS) served as the reference (Yang et al., 2020). Cell viability was calculated as a percentage relative to the vehicle-treated control group.

### Osteoclast Formation Assay

For osteoclast differentiation assessment *in vitro*, we seeded RAW264.7 or BMM cells at a density of 2.0 × 10^3^ cells/well in triplicate in medium containing varying concentrations of CIM (0, 80, 160, 320 μM) and 50 ng/ ml RANKL. The medium was replaced every 24 h for 5–7 days until multinuclear giant cells were clearly observed. The cells were then rinsed twice with PBS and fixed with 4% paraformaldehyde for 20 min. After removing the paraformaldehyde solution, the TRAP staining procedure was conducted according to the manufacturer’s instructions. TRAP-positive cells with more than three nuclei were counted under a microscope, and the images were captured for measurements using ImageJ software (NIH, Bethesda, MD, United States).

### Resorption Pit Formation Assay

Bovine bone slices were placed in the wells of 96-well plates after being sterilized and dried. RAW264.7 cells (2.0 × 10^3^ cells/well) were seeded at 2.5 × 10^3^ cells/well onto the bone slices in triplicate and treated with 50 ng/ml RANKL and CIM (0, 80, 160, 320 μM) for 7 days until mature osteoclasts formed. Then, the cells on the bone slices were removed by brushing and sonication. The osteolytic surfaces of bone slices were visualized by scanning and imaging with a scanning electron microscope (SEM; FEI Quanta 250). The captured images were analyzed using ImageJ software for the evaluation of bone resorption.

### F-Actin Ring Immunofluorescence Assay

To further quantify the effect of CIM on osteoclast activity, BMMs were treated with 50 ng/ ml RANKL and different concentrations of CIM (0, 80, 320 μM); this protocol was identical to the previously described cell preparation procedure performed prior to TRAP staining in the osteoclast formation assay. Cell samples were permeabilized with PBS containing 0.1% Triton X (Sigma Aldrich, St Louis, MO, United States) for 5 min. Then, the cells were incubated with Alexa Fluor 647 phalloidin (Invitrogen, San Diego, CA, United States) diluted in 0.2% (w/v) BSA-PBS (Invitrogen, San Diego, CA, United States) for 1 h in the dark. After extensive rinsing with PBS, the cells were mounted with ProLong Gold anti-fade mounting medium (Invitrogen, San Diego, CA, United States) to visualize cell nuclei (Zhu et al., 2019). We used a NIKON A1Si spectral detector confocal system (Nikon, Tokyo, Japan) with NISC Elements software to acquire fluorescence images, and the F-actin-positive cells were quantified using ImageJ software.

### RNA Isolation and Quantitative Real-Time Polymerase Chain Reaction (qRT–PCR) Analysis

qRT–PCR analysis was performed to assess the effect of CIM on osteoclast differentiation at the transcriptional level. BMMs (1.0 × 10^5^ cells/well) were seeded in 24-well plates in triplicate in c-α-MEM supplemented with 30 ng/ ml M-CSF, 50 ng/ ml RANKL and different doses of CIM (0, 80, 160, 320 μM) for 5 days. Total RNA was extracted from the different groups of cells with the Qiagen RNeasy Mini kit (Qiagen; Valencia, CA, United States) according to the manufacturer’s instructions. Then, 1 mg of total RNA was reverse transcribed using a reverse transcriptase kit (TaKaRa Biotechnology; Otsu, Japan) to produce cDNAs (complementary DNAs), which served as the templates for the subsequent qRT–PCR assays using a SYBR Premix Ex Taq kit (TaKaRa Biotechnology) and an ABI 7500 sequencing detection system (Applied Biosystems; Foster City, CA, United States). qRT–PCR was conducted for 40 cycles of denaturation at 95°C for 5 s and amplification at 60°C for 34 s, and the levels of osteoclast-specific genes were normalized to the internal reference Gapdh. We analyzed the data with the 2 (-delta delta C(T)) method (Livak method) (Zhang et al., 2018). The murine primer sequences are listed in Table 1.

**TABLE 1:** Murine primer sequences for quantitative real-time PCR analysis.

Gene	Forward primer (5’-3’)	Reverse primer (3’-5’)
c-Fos	CCA​GTC​AAG​AGC​ATC​AGC​AA	AAG​TAG​TGC​AGC​CCG​GAG​TA
Traf6	AAA​CCA​CGA​AGA​GGT​CAT​GG	GCG​GGT​AGA​GAC​TTC​ACA​GC
Acp5	TCC​TGG​CTC​AAA​AAG​CAG​TT	ACA​TAG​CCC​ACA​CCG​TTC​TC
Calcr	CGG​ACT​TTG​ACA​CAG​CAG​AA	AGC​AGC​AAT​CGA​CAA​GGA​GT
Ctsk	CTT​CCA​ATA​CGT​GCA​GCA​GA	TCT​TCA​GGG​CTT​TCT​CGT​TC
Nfatc1	CCG​TTG​CTT​CCA​GAA​AAT​AAC​A	TGT​GGG​ATG​TGA​ACT​CGG​AA
Dc-stamp	AAA​ACC​CTT​GGG​CTG​TTC​TT	AAT​CAT​GGA​CGA​CTC​CTT​GG
Gapdh	ACC​CAG​AAG​ACT​GTG​GAT​GG	CAC​ATT​GGG​GGT​AGG​AAC​AC

### Western Blot Analysis

To elucidate the effect of CIM on RANKL-induced osteoclast differentiation, we seeded RAW264.7 cells at 5.0 × 10^5^ cells/well in 6-well plates. After reaching confluence, the cells were pretreated with α-MEM containing 320 μM CIM or not for 4 h and then stimulated with α-MEM containing 50 ng/ ml RANKL for 0, 5, 10, 20, 30, or 60 min. The total proteins were isolated using radioimmunoprecipitation assay (RIPA) lysis buffer (Beyotime, Shanghai, China) supplemented phenylmethylsulfonyl fluoride (PMSF) followed by centrifugation at 12,000 rpm for 15 min. The supernatant enriched with protein products was collected and quantified with a bicinchoninic acid assay kit (Biosharp Life Science, China) according to the manufacturer’s instructions. Next, the protein products (30 mg) from each group were separated by sodium dodecyl sulfate polyacrylamide gel electrophoresis (SDS–PAGE) on 10% gels and then transferred onto PVDF membranes (Millipore, Bedford, MA, United States). After being blocked with 5% (w/v) skimmed milk powder in TBS-Tween (Tris-buffered saline-Tween: 0.1% Tween-20 in TBS) at room temperature for 1 h, the PVDF membranes were incubated for 12 h at 4°C with primary antibodies targeting nuclear factor of kappa light polypeptide gene enhancer in B-cells inhibitor alpha (IκBα), p-IκBα, JNK, p-JNK, ERK, p-ERK, p38, p-p38, and β-actin (Cell Signaling Technology, Cambridge, MA, United States). Then, the membranes were rinsed and incubated with the corresponding secondary antibodies at room temperature for 1 h. Antibody reactivity and protein bands were detected based on exposure with an Odyssey V3.0 imaging system (LI-COR, Lincoln, NE, United States). The band intensities were quantified using ImageJ software.

### Luciferase Reporter Gene Assay

To further investigate the inhibitory effect of CIM on the RANKL-induced activation of the NF-κB signaling pathway, the NF-κB luciferase reporter 3 kB-Luc-SV40 was constructed and stably transfected into RAW264.7 cells as described previously (Zhai et al., 2014). The cells were then seeded into 48-well plates, incubated for 24 h to allow adhesion and subsequently treated with CIM at concentrations of 0, 80, 320 μM. Then, 50 ng/ ml RANKL was administered to each well for another 8 h. Luciferase activity was determined based on the fluorescence intensity detected with the Promega luciferase Assay System (Promega, Madison, WI, United States), and the vehicle group served as the control (Ikeda et al., 2004).

### Ti Particle-Induced Murine Calvarial Osteolysis Model

Ti particles with an average diameter of 4.5 μm were purchased from Johnson Matthey (Ward Hill, MA, United States). To remove endotoxins, the particles were baked for 6 h at 180°C and then immersed in 75% ethanol. The mixture was shaken on a horizontal shaker for 24 h. Ti particles were resuspended in sterile PBS at 0.3 g /ml and confirmed to be free of endotoxins via a Limulus amebocyte lysate assay before being stored at 4°C. Animal experimental protocols were performed in accordance with the recommendations of the Animal Care Committee of Central South University and were approved by the Animal Care Committee of Central South University.

Eighteen 6- to 8-week-old C57BL/6 male mice (weight: 22.64 ± 1.54 g) were acquired from Shanghai Slac Laboratory Animal Company and raised in three specific pathogen-free (SPF) cages with six mice in each group. The groups were as follows: mice that underwent a sham operation and were treated with PBS (sham group), those implanted with Ti particles and were treated with PBS (vehicle group), and those implanted with Ti particles and were treated with 50 mg/ kg CIM (CIM group). Before the surgery, the mice were anesthetized with an intraperitoneal injection of 4% chloral hydrate at 0.1 ml/10 g∙bw. The cranial periosteum was cleaved via a median incision and sutured with 30 mg of Ti particles embedded between the periosteum and the calvarium (Wu et al., 2015). After the first 2 days, the mice in the CIM group were intragastrically administered 50 mg/ kg CIM every 2 days, and mice in the sham and vehicle groups received PBS for 8 weeks. Finally, the mice were euthanized 2 days after the last treatment for the harvesting of calvaria samples, which were fixed with 4% paraformaldehyde and analyzed by microcomputed tomography (micro-CT).

### Micro-CT Scanning

A high-resolution micro-CT system (μCT50; Scanco; Zurich; Switzerland) was employed to assess the morphology of calvarial osteolysis. The scanning was conducted with the following parameters: 10 μm isometric resolution, 300 ms exposure time, and 80 μA X-ray energy. The reconstructed images of the calvarial surface around the median incision were obtained as the region of interest, and the morphological parameters, including the bone mineral density (BMD), bone volume versus tissue volume (BV/TV), number of pores, and percentage of total porosity, were analyzed.

### Immunohistochemical Staining and Histomorphometric Analysis

After micro-CT scanning, the fixed calvaria samples were decalcified with 10% EDTA for 3 weeks and embedded in paraffin. Then, the samples were sectioned serially and subjected to TRAP and H and E staining. The images were collected by a high-powered microscope, and TRAP-positive multinucleated osteoclasts were quantified using ImageJ software.

### Statistical Analysis

Experimental data were acquired from at least three replicates and are presented as the means ± SDs (standard deviations). Data analyses were performed using one-way analysis of variance (ANOVA) followed by Dunnett’s *post hoc* test to compare group differences with SPSS 20.0 software (SPSS Inc., United States). Significant differences between the different groups are indicated by * for *p* < 0.05 and by ** for *p* < 0.01.

## Results

### CIM at a Noncytotoxic Dose Repressed Osteoclastogenesis *in Vitro*

**FIGURE 1:**
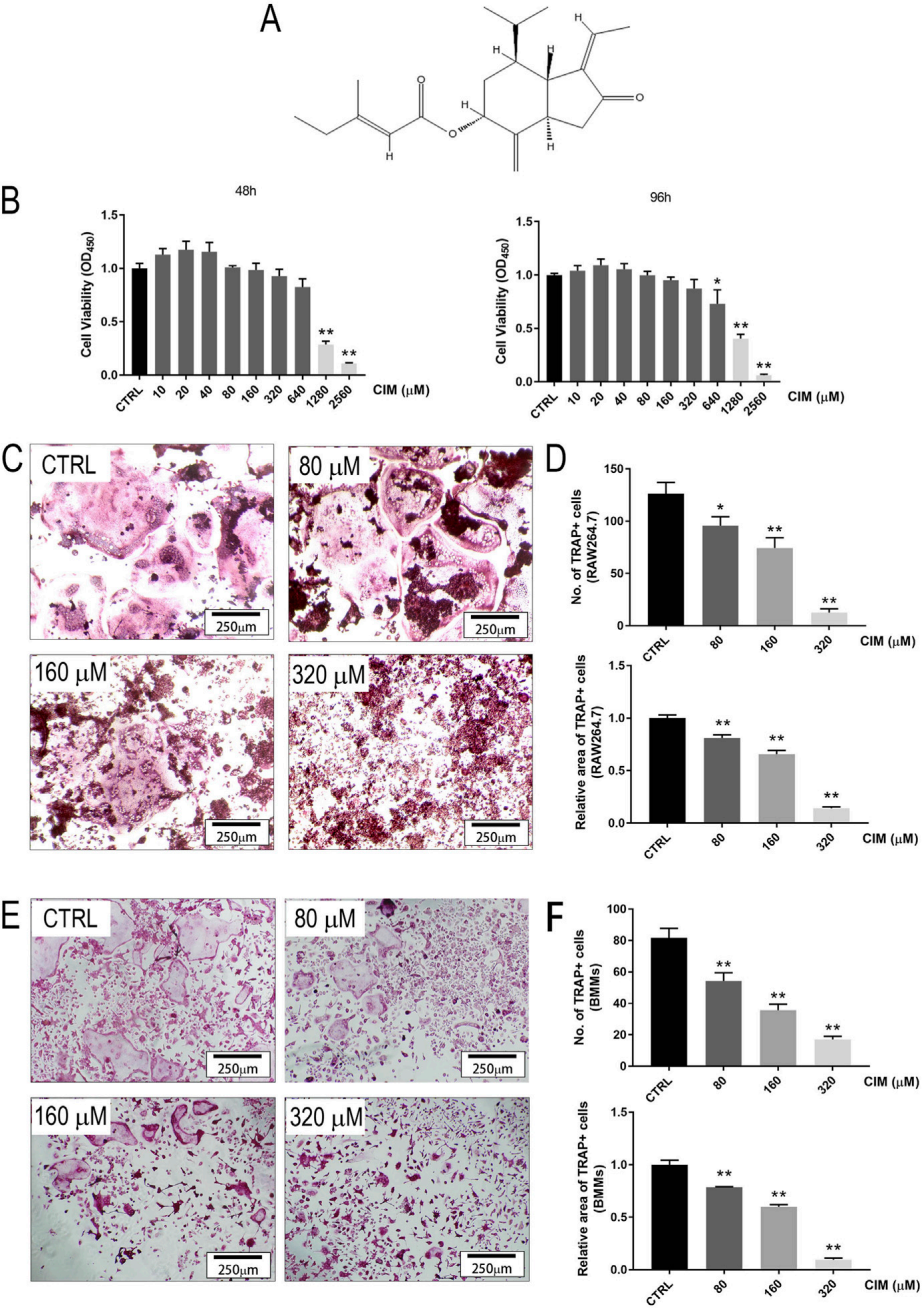
CIM at a noncytotoxic dose inhibits RANKL-induced osteoclast formation in RAW264.7 and BMM cell lineages in a dose-dependent manner. **(A)** The chemical structure of CIM. **(B)** The cell viability of BMMs treated with CIM at different doses for 48 or 96 h. CIM at doses less than or equal to 320 μM was identified as noncytotoxic. **(C) and (E)** TRAP staining images of RAW264.7 cells and BMMs incubated with CIM at gradient concentrations for 5–7 days. The numbers **(D)** and areas **(F)** of TRAP + osteoclasts were quantified and analyzed. (*: *p* < 0.05; **: *p* < 0.01 compared with the control group).

The chemical structural formula of CIM is presented in Figure 1A. To avoid the cytotoxic effect of CIM on osteoclast formation, the noncytotoxic concentration of CIM in osteoclast precursors was identified. The results suggested that CIM at concentrations greater than 640 μM significantly reduced the number of osteoclast precursors, while CIM at concentrations below 320 μM did not, indicating a noncytotoxic threshold of 320 μM for the subsequent osteoclast formation assays (Figure 1B **)**.

Then, the inhibitory effects of CIM on RANKL-induced osteoclast formation *in vitro* were investigated. As shown in Figures 1C,E, excessive numbers of TRAP + mature multinucleated giant cells with distinct cytoplasms, identified as osteoclasts, were observed in both RAW264.7 and BMM cell lineages. However, the numbers and areas of osteoclasts in groups treated with CIM at varying concentrations were dose-dependently decreased (Figures 1C-E). CIM applied at 320 μM significantly inhibited osteoclastogenesis, especially in the RAW264.7 group.

### CIM Attenuated the Bone-Resorbing Function of Mature Osteoclasts *in Vitro*

**FIGURE 2:**
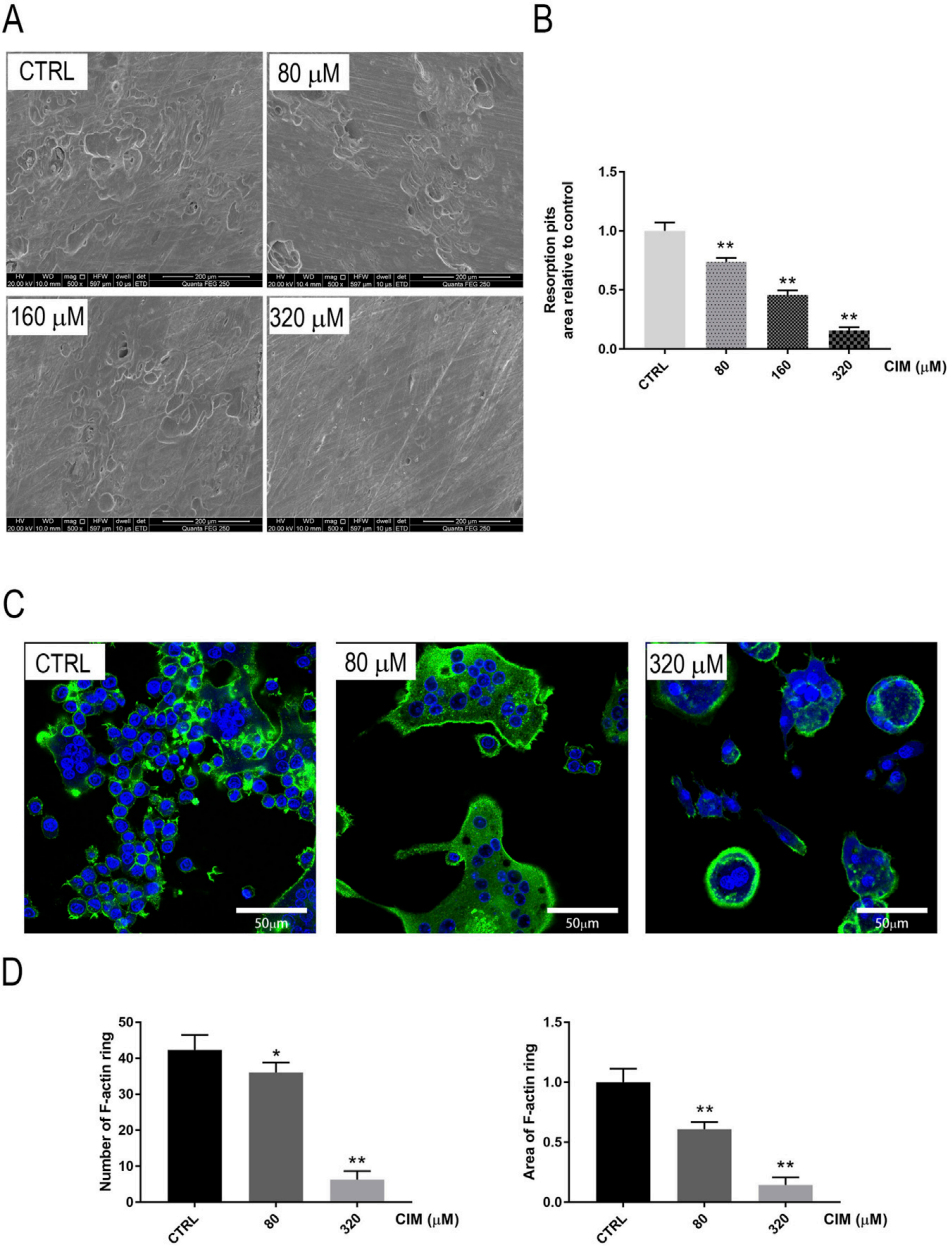
Noncytotoxic CIM dose-dependently mitigates the bone-resorbing activity of osteoclasts *in vitro*. **(A)** Scanning electron microscopy images of eroded surfaces on bone slices treated with CIM at the indicated concentrations. **(B)** The areas of bone resorption pits relative to the control group are shown. **(C)** Immunofluorescence images of F-actin rings (in green) in BMM-derived osteoclasts treated with CIM at the indicated doses. (**: *p* < 0.01 compared with the control group). **(D)** Number and area of F-actin ring were counted *via* Image J Software.

Given the inhibitory effect of CIM on osteoclastogenesis, we assumed that CIM would decrease the bone-resorbing function of osteoclasts in parallel. To prove this, a bone slice resorption assay was performed. As demonstrated in Figures 2A,B, RANKL-induced resorption pit formation was clearly observed on the surface in the control group, while CIM treatment decreased the resorption pit area, as expected. According to the quantified results, the resorption pit area was decreased by approximately 85% relative to the control after treatment with 320 μM CIM.

The F-actin ring in the cytoplasm is widely recognized as an indicator of functional osteoclasts in bone resorption (Holliday et al., 2019). Therefore, we quantified F-actin in CIM-treated osteoclasts using an immunofluorescence assay. As shown in Figures 2C,D, F-actin rings were well polarized together with RANKL-induced osteoclastogenesis, while CIM treatment reduced the number and area of F-actin rings, which further proved its ability to repress the functions of osteoclasts. In summary, these data suggested that CIM at a noncytotoxic concentration inhibits osteoclast formation and bone-resorbing function.

### IM Inhibits the Expression of Osteoclast-specific Genes *in Vitro*

**FIGURE 3:**
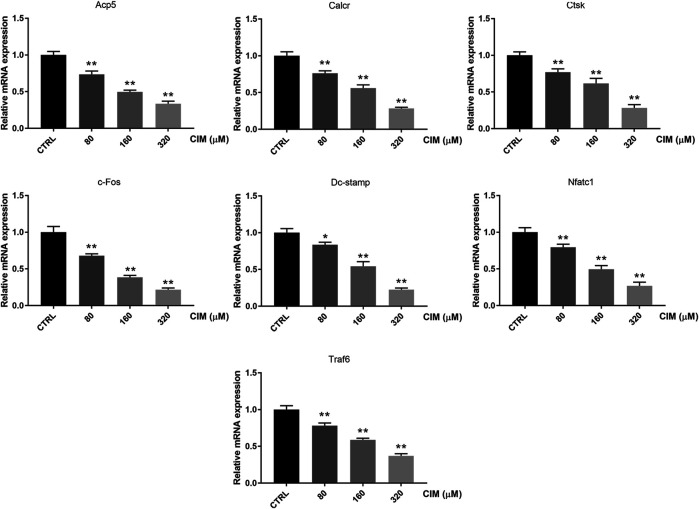
CIM dose-dependently represses the expression of osteoclast marker genes, including c-Fos, Nfatc1, Acp5, Calcr, Ctsk, Dc-stamp and Traf6, in RANKL-stimulated osteoclasts. These genes were quantified by qRT–PCR and normalized to the level of the housekeeping Gapdh. (*: *p* < 0.05; **: *p* < 0.01 compared with the control group).

RANKL activates the excessive expression of specific genes and the subsequent osteoclast differentiation. Therefore, we performed qRT–PCR to examine the effect of CIM on osteoclast-specific genes. As indicated in Figure 3, the expression of specific genes encoding key factors and markers of osteoclastogenesis, including c-Fos, Nfatc1, Acp5, Calcr, Ctsk, Dc-stamp, and Traf6, was dose-dependently inhibited by CIM treatment. These results further supported the repressive effect of CIM on osteoclastogenesis and bone-resorbing function.

### CIM Alleviated RANKL-Induced NF-κB Signaling in Osteoclastogenesis *in Vitro*

**FIGURE 4:**
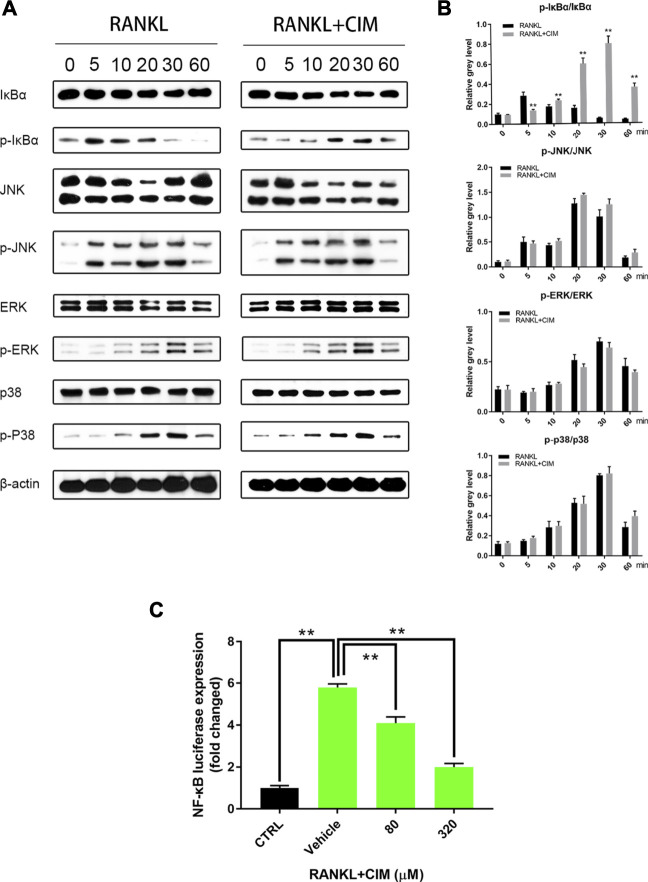
CIM downregulates the NF-κB signaling pathway but not the MAPK signaling in RANKL-pretreated osteoclast precursors. **(A)** Chronological Western blot band spectra corresponding to key factors and their phosphorylated forms in the NF-κB and MAPK (JNK, ERK and p38) signaling pathways in BMMs pretreated with 320 μM CIM and RANKL are presented; the levels were compared to those in the control group (only RANKL). **(B)** The levels of phosphorylated NF-κB and MAPK proteins were normalized to their total levels and analyzed. **(C)** The luciferase activity of RAW264.7 cells transfected with the NF-κB luciferase reporter gene was quantified. (**: *p* < 0.01 compared with the control group).

To elucidate the molecular mechanism underlying the antiosteoclastogenic activity of CIM, Western blotting was performed to assess the time-dependent expression of key factors mediating the NF-κB and MAPK (JNK, ERK, and p38) signaling cascades in RANKL-induced osteoclastogenesis. As reflected by the serial bands shown in Figures 4A,B, p-IκBα expression peaked within 5 min in the control group. However, CIM pretreatment repressed the level of p-IκBα at 5–10 min and delayed the phosphorylation peak from 5 to 30 min, suggesting that the activation of NF-κB was significantly inhibited by CIM. Moreover, the luciferase reporter assay further demonstrated that CIM impaired NF-κB (Figure 4C). In the MAPK signaling cascade, the phosphorylation levels of JNK, ERK, and p38 were similar between the control and CIM-pretreated groups, indicating that RANKL-induced MAPK signaling activation was not affected by CIM. Collectively, these results proved that CIM inhibited osteoclastogenesis by attenuating NF-κB signaling without significantly affecting the MAPK (JNK, ERK, and p38) signaling pathway.

### CIM Treatment Attenuated Ti Particle-Induced Osteolysis *in Vivo*

**FIGURE 5:**
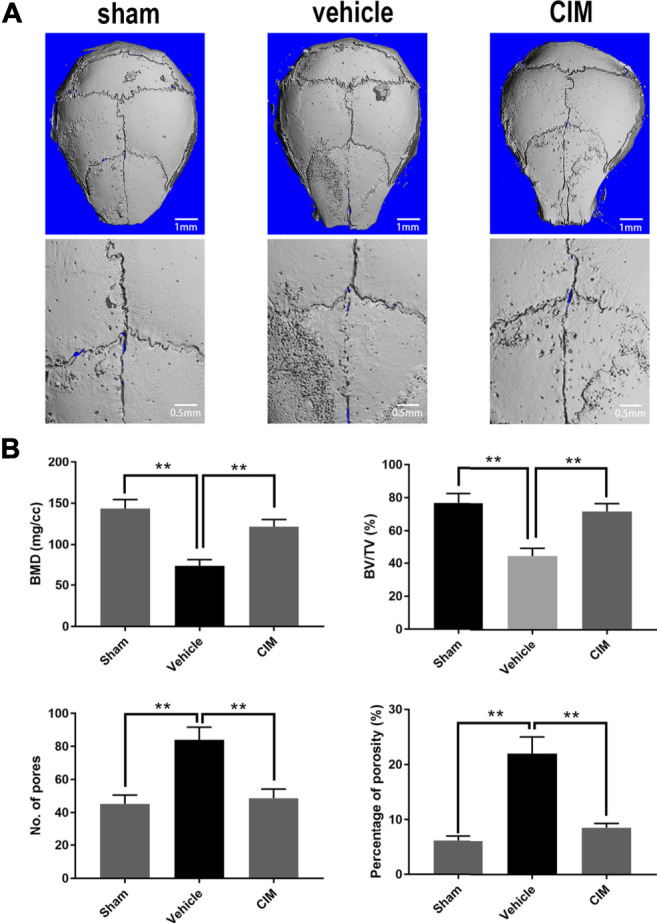
CIM protects murine calvaria from Ti particle-induced osteolysis *in vivo*. **(A)** Reconstructed micro-CT radiograph of harvested murine calvaria. **(B)** The bone mineral density (BMD), bone volume versus tissue volume (BV/TV), number of pores and percentage of total porosity within regions of interest were analyzed. (**: *p* < 0.01 versus the control group).

Based on the above results, we verified the therapeutic effect of CIM on ALP in a Ti particle-induced murine calvarial osteolysis model. As demonstrated by the micro-CT scanning shown in Figure 5, the bone erosion observed in the vehicle group was extensive compared to that in the control group, whereas intragastrical CIM treatment significant attenuated the severity of calvarial osteolysis in regards to multiple bone morphometric indices, including the BMD, BV/TV, number of pores and percentage of porosity.

**FIGURE 6:**
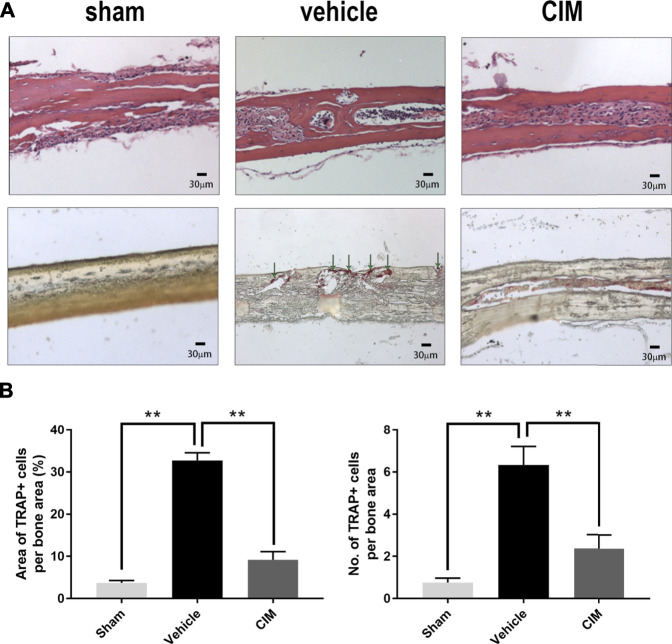
Histological staining showing inhibited bone erosion and osteoclast accumulation in the harvested murine calvaria. **(A)** Representative images of H and E and TRAP staining ( × 100) in the indicated groups (the green arrows refer to the area of TRAP + cells). **(B)** Diagram and statistical analysis of the area and number of TRAP + cells per bone region. (**: *p* < 0.01 versus the control group).

Similarly, images of H and E- and TRAP-stained samples (Figure 6) further demonstrated remarkable osteolysis in the slice cross sections and the accumulation of TRAP + osteoclasts in the vehicle group. Consistent with the morphometric changes observed by micro-CT imaging, CIM administration considerably protected the calvaria from bone erosion by reducing osteoclasts. Taken together, the data from the animal experiments further support that CIM has potential as a therapeutic for Ti particle-induced osteolysis due to its ability to inhibit osteoclastogenesis.

## Discussion

Nearly 2.5 million patients who suffer from joint dysfunction caused by diseases such as hip and knee osteoarthritis undergo arthroplasty surgery each year worldwide (Nemeth et al., 2021). Artificial joint replacement has gained ground as one of the most effective treatments for articular diseases for which conservative approaches fail. This number will continue to rise over the next 20–30 years for younger patients who are prone to higher exercise loads. Moreover, approximately 5% of patients undergo revision surgery due to ALP within the first 15 years after primary arthroplasty surgery (Goodman and Gallo, 2019). Thus, the burden of ALP secondary to periprosthetic osteolysis is increasing worldwide. ALP is one of the most common long-term complications of arthroplasty. It is widely accepted that wear particle-induced osteolysis is the leading cause of ALP after arthroplasty (Howie et al., 2013). Specifically, wear particles are phagocytosed by accumulating macrophages and then trigger the overexpression of inflammatory mediators such as IL-1, IL-6, IL-17, TNF-α, M-CSF, monocyte chemoattractant factor 1 (MCP-1), and macrophage inflammatory protein 1 alpha (MIP-1α), which promote the differentiation of macrophages into osteoclasts (Dyskova et al., 2017; Gibon et al., 2017). RANKL, a specific receptor activator for the NF-κB ligand expressed by osteoblasts and osteocytes, serves as another key molecule in osteoclastogenesis (Goodman and Gallo, 2019). Both of these factors could cause periprosthetic osteolysis and subsequent ALP (Howie et al., 2013; Lin et al., 2014). Given the improvements in material and manufacturing technology, ALP seems to be inevitable. To date, nearly all types of debris from different interfaces of the prosthesis system, including metal, polymethyl methacrylic (PMMA), polyethylene (PE) and ceramics, have been reported as causes of periprosthetic osteolysis of varying severity (Merkel et al., 1999; Gibon et al., 2017). Therefore, pharmacotherapies targeting osteoclasts have attracted substantial attention.

**FIGURE 7:**
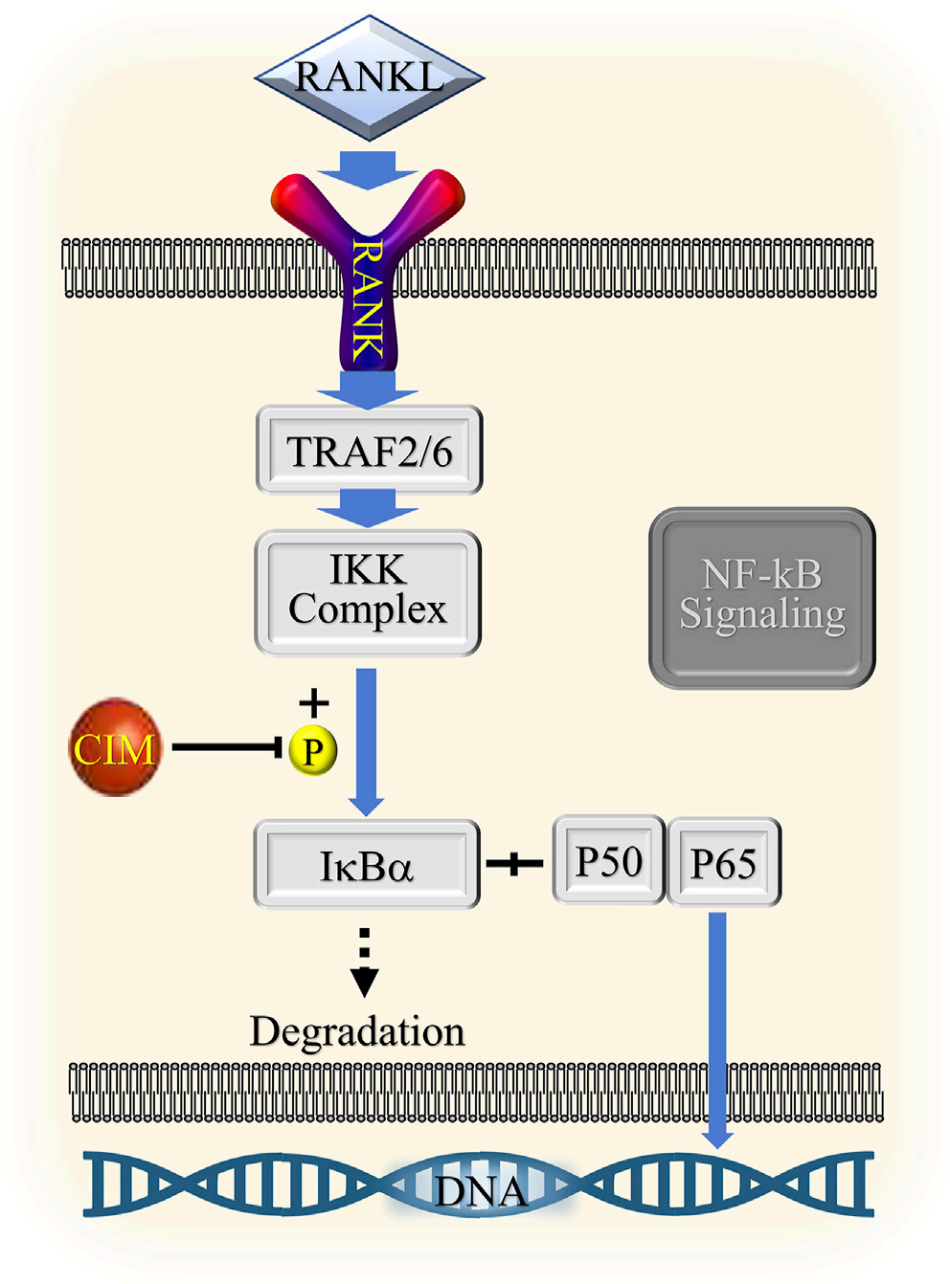
Schematic diagram of the presumed mechanism by which CIM inhibits NF-κB signaling.

In the current study, we demonstrated the inhibitory effect of CIM on osteoclastogenesis *in vitro*. CIM at a noncytotoxic dose repressed osteoclast differentiation in RAW264.7 cells and BMMs and alleviated subsequent bone resorption. The impaired formation of the F-acting ring after CIM treatment also confirmed the above findings. Moreover, we discovered that CIM downregulated the expression of specific genes in osteoclasts, including those involved in the regulation of downstream gene expression (Nfatc1, c-Fos and Traf6) (Park et al., 2017), bone resorption function (Ctsk and Acp5) (Walia et al., 2018), calcium homeostasis (Calcr) (Granholm et al., 2011), and cell fusion of precursors (Dc-stamp) (Kodama and Kaito, 2020). Then, we further proved that CIM protected bone from Ti particle-induced osteolysis *in vivo*. This potential therapeutic effect of CIM on ALP was supported by the results of both micro-CT scanning and immunohistochemical assays. After confirming the antiosteoclastogenic activity of CIM, we elucidated its potential molecular mechanism. RANKL specifically binds to RANK on the cytomembrane of osteoclast precursors and then triggers the recruitment of TRAFs and TAK1 binding protein two in the cytoplasm (Asagiri and Takayanagi, 2007). The RANKL/RANK/TRAF complex then activates the phosphorylation of TGF-β-activated kinase 1 (TAK1), which in turn initiates both NF-κB and MAPK signaling by phosphorylating both the IKK complex and MKKs (Asagiri and Takayanagi, 2007; Sun et al., 2020). First, the phosphorylated IKK complex induces the cleavage of NF-κB from IκB and the subsequent degradation of IκB (Wada et al., 2006). In addition, phosphorylated MKKs activate the phosphorylation of JNK, ERK and p38 (Feng, 2005). The activation of all these signaling pathways contribute to the upregulation of osteoclast-specific gene expression. In the current study, we found that CIM inhibited osteoclastogenesis by downregulating the phosphorylation of IκBα in the NF-κB signaling pathway (Figure 7).

The CIM chromone was first discovered in Cimicifuga racemosa but is mainly prepared from the root of *Saposhnikovia divaricata*; both of these species have long been used in traditional Chinese medicines to treat upper respiratory infections and skin inflammatory diseases. Previous studies have proven that CIM has the potential to treat multiple inflammatory diseases. Liu et al. (Liu et al., 2020) reported that CIM could mitigate imiquimod-induced psoriasis in mice by inhibiting oxidative stress, inhibiting inflammation and repressing NF-κB and MAPK signaling in HaCaT cells. Han et al. (2019) concluded that CIM could alleviate lipopolysaccharide-induced inflammatory responses in RAW264.7 cells as a rheumatoid arthritis model by inhibiting the phosphorylation of IκB, ERK and p38 (without affecting JNK). In the present study, we demonstrated that CIM inhibited RANKL-induced NF-κB signaling by blocking the phosphorylation of IκBα without affecting JNK, ERK or p38 activation in the MAPK signaling cascade during osteoclastogenesis. This discrepancy may be due to the diversity of the cell and animal models utilized and to the limited exploration of molecular mechanisms. Imiquimod and lipopolysaccharide were adopted by Liu et al. and Han et al. to establish experimental models, as they share identical pathways downstream of TRAF6 with RANKL-induced signaling but not upstream of TRAF6. CIM treatment may simultaneously alter the regulatory pattern upstream of TRAF6 and the NF-κB pathway, accounting for the differential conclusions drawn by the authors. In addition, time-dependent changes in NF-κB and MAPK signaling proteins were not reported in the studies by Liu et al. and Han et al. Moreover, all these studies, including the present study, investigated the levels of only a limited number of signaling proteins, and further studies may thus be needed to elucidate the mechanism of cimifugin in osteoclasts.

This study does have several limitations. First, we assessed the expression of only IκB, JNK, ERK, p38 and their phosphorylated forms as key factors in the NF-κB and MAPK signaling pathways. To elucidate the exact target proteins in the cascades and the sites at which the specific proteins act, more experiments, including Western blot analyses of upstream signaling proteins, “rescue” experiments and molecular docking assays, may be required. Second, homeostasis of bone metabolism inevitably involves bone resorption induced by osteoclasts and bone formation induced by osteoblasts. The effect of CIM on osteoblast-induced bone formation should be explored in future studies. Finally, the adopted Ti particle-induced murine calvarial osteolysis model does not perfectly simulate ALP. From metal and ultrahigh-molecular-weight polyethylene (UHMWPE) to ceramic and polyether-ether-ketone (PEEK), the particles worn by interfaces of any kind could contribute to periprosthetic osteolysis of varying severity (Wooley, 2014). As the cortical bone is thin and bears little stress, the murine calvarium serves as only a defective analog of ALP in the hip or knee joint of humans (Hu et al., 2020). However, this model still exhibits pathological changes similar to those of the polyethylene particle-induced model and is widely used in studies of ALP (Taki et al., 2005; Sun et al., 2020). This study also provides limited new insight into the lack of biocompatibility of cimifugin compared with the currently available clinical drugs. However, no significant body weight loss, death or adverse effects were observed either control group or CIM group animals. Therefore, the biosafety of CIM in mice was seemingly acceptable in this preliminary study, and further investigations with more detailed parameters related to biocompatibility and tissue specificity should be carried out in the future.

Conclusively, we demonstrated for the first that CIM alleviate RANKL-induced osteoclastogenesis and Ti particle-induced osteolysis *in vitro* and *in vivo* by inhibiting the NF-κB signaling pathway. The current findings suggest the potential of CIM as a treatment for ALP as well as other osteopathies mediated by excessive osteoclasts, thereby broadening the spectrum of bone-protective natural compounds.

## Data Availability

The original contributions presented in the study are included in the article/Supplementary Material, further inquiries can be directed to the corresponding authors.

## Abbreviations

ALP, Aseptic loosening of prosthesis; BMMs, bone marrow-derived macrophages; BV, bone volume; CIM, cimifugin; c-α-MEM, complete α-MEM; MAPKs, mitogen-activated protein kinases; NF-κB, nuclear factor κ B; PBS, phosphate-buffered saline; qRT–PCR, polymerase chain reaction; RANKL, receptor activator of nuclear factor κ B ligand; TRAP, tartrate-resistant acid phosphatase; TV, tissue volume; TRAF6, TNF receptor-associated factor 6.

## References

[B1] Asagiri M. Takayanagi H. (2007). The Molecular Understanding of Osteoclast Differentiation. Bone 40 (2), 251–264. 10.1016/j.bone.2006.09.023 17098490

[B2] Dyskova T. Gallo J. Kriegova E. (2017). The Role of the Chemokine System in Tissue Response to Prosthetic By-Products Leading to Periprosthetic Osteolysis and Aseptic Loosening. Front. Immunol. 8, 1026. 10.3389/fimmu.2017.01026 28883822 PMC5573717

[B3] Feng X. (2005). RANKing Intracellular Signaling in Osteoclasts. IUBMB Life 57 (6), 389–395. 10.1080/15216540500137669 16012047

[B4] Gibon E. Córdova L. A. Lu L. Lin T. H. Yao Z. Hamadouche M. (2017). The Biological Response to Orthopedic Implants for Joint Replacement. II: Polyethylene, Ceramics, PMMA, and the Foreign Body Reaction. J. Biomed. Mater. Res. B Appl. Biomater. 105 (6), 1685–1691. 10.1002/jbm.b.33676 27080740 PMC5536115

[B5] Goodman S. B. Gallo J. (2019). Periprosthetic Osteolysis: Mechanisms, Prevention and Treatment. J. Clin. Med. 8 (12), 2091. 10.3390/jcm8122091 31805704 PMC6947309

[B6] Granholm S. Henning P. Lerner U. H. (2011). Comparisons between the Effects of Calcitonin Receptor-Stimulating Peptide and Intermedin and Other Peptides in the Calcitonin Family on Bone Resorption and Osteoclastogenesis. J. Cel Biochem 112 (11), 3300–3312. 10.1002/jcb.23256 21748786

[B7] Han B. Dai Y. Wu H. Zhang Y. Wan L. Zhao J. (2019). Cimifugin Inhibits Inflammatory Responses of RAW264.7 Cells Induced by Lipopolysaccharide. Med. Sci. Monit. 25, 409–417. 10.12659/msm.912042 30638197 PMC6342062

[B8] He K. Zheng B. Kim C. H. Rogers L. Zheng Q. (2000). Direct Analysis and Identification of Triterpene Glycosides by LC/MS in Black Cohosh, Cimicifuga Racemosa, and in Several Commercially Available Black Cohosh Products. Planta Med. 66 (7), 635–640. 10.1055/s-2000-8619 11105569

[B9] Holliday L. S. Faria L. P. Rody W. J. Jr. (2019). Actin and Actin-Associated Proteins in Extracellular Vesicles Shed by Osteoclasts. Int. J. Mol. Sci. 21 (1), 158. 10.3390/ijms21010158 31881680 PMC6981389

[B10] Howie D. W. Neale S. D. Haynes D. R. Holubowycz O. T. McGee M. A. Solomon L. B. (2013). Periprosthetic Osteolysis after Total Hip Replacement: Molecular Pathology and Clinical Management. Inflammopharmacology 21 (6), 389–396. 10.1007/s10787-013-0192-6 24127125

[B11] Hu X. Yin Z. Chen X. Jiang G. Yang D. Cao Z. (2020). Tussilagone Inhibits Osteoclastogenesis and Periprosthetic Osteolysis by Suppressing the NF-Κb and P38 MAPK Signaling Pathways. Front. Pharmacol. 11, 385. 10.3389/fphar.2020.00385 32317967 PMC7146087

[B12] Ikeda F. Nishimura R. Matsubara T. Tanaka S. Inoue J. Reddy S. V. (2004). Critical Roles of C-Jun Signaling in Regulation of NFAT Family and RANKL-Regulated Osteoclast Differentiation. J. Clin. Invest. 114 (4), 475–484. 10.1172/jci19657 15314684 PMC503767

[B13] Jia Z. Tie C. Wang C. Wu C. Zhang J. (2019). Perturbed Lipidomic Profiles in Rats with Chronic Cerebral Ischemia Are Regulated by Xiao-Xu-Ming Decoction. Front. Pharmacol. 10, 264. 10.3389/fphar.2019.00264 30941043 PMC6433774

[B14] Kim J. M. Lin C. Stavre Z. Greenblatt M. B. Shim J. H. (2020). Osteoblast-Osteoclast Communication and Bone Homeostasis. Cells 9 (9), 2073. 10.3390/cells9092073 32927921 PMC7564526

[B15] Kodama J. Kaito T. (2020). Osteoclast Multinucleation: Review of Current Literature. Int. J. Mol. Sci. 21 (16), 5685. 10.3390/ijms21165685 32784443 PMC7461040

[B16] Lin T. H. Tamaki Y. Pajarinen J. Waters H. A. Woo D. K. Yao Z. (2014). Chronic Inflammation in Biomaterial-Induced Periprosthetic Osteolysis: NF-Κb as a Therapeutic Target. Acta Biomater. 10 (1), 1–10. 10.1016/j.actbio.2013.09.034 24090989 PMC3849197

[B17] Liu A. Zhao W. Zhang B. Tu Y. Wang Q. Li J. (2020). Cimifugin Ameliorates Imiquimod-Induced Psoriasis by Inhibiting Oxidative Stress and Inflammation via NF-Κb/MAPK Pathway. Biosci. Rep. 40 (6), BSR20200471. 10.1042/bsr20200471 32515468 PMC7300284

[B18] Merkel K. D. Erdmann J. M. McHugh K. P. Abu-Amer Y. Ross F. P. Teitelbaum S. L. (1999). Tumor Necrosis Factor-Alpha Mediates Orthopedic Implant Osteolysis. Am. J. Pathol. 154 (1), 203–210. 10.1016/s0002-9440(10)65266-2 9916934 PMC1853441

[B19] Nemeth B. Nelissen R. Arya R. Cannegieter S. (2021). Preventing VTE Following Total Hip and Knee Arthroplasty: Is Prediction the Future? J. Thromb. Haemost. 19 (1), 41–45. 10.1111/jth.15132 33043553 PMC7839761

[B20] Ouyang Z. Zhai Z. Li H. Liu X. Qu X. Li X. (2014). Hypericin Suppresses Osteoclast Formation and Wear Particle-Induced Osteolysis via Modulating ERK Signalling Pathway. Biochem. Pharmacol. 90 (3), 276–287. 10.1016/j.bcp.2014.06.009 24950468

[B21] Panegrossi G. Ceretti M. Papalia M. Casella F. Favetti F. Falez F. (2014). Bone Loss Management in Total Knee Revision Surgery. Int. Orthop. 38 (2), 419–427. 10.1007/s00264-013-2262-1 24407821 PMC3923937

[B22] Park J. H. Lee N. K. Lee S. Y. (2017). Current Understanding of RANK Signaling in Osteoclast Differentiation and Maturation. Mol. Cell 40 (10), 706–713. 10.14348/molcells.2017.0225 29047262 PMC5682248

[B23] Prock-Gibbs H. Pumilia C. A. Meckmongkol T. Lovejoy J. Mumith A. Coathup M. (2021). Incidence of Osteolysis and Aseptic Loosening Following Metal-On-Highly Cross-Linked Polyethylene Hip Arthroplasty. J. Bone Jt. Surg Am Publish Ahead Print 103, 728–740. 10.2106/jbjs.20.01086 33411465

[B24] Sun G. J. Yang S. F. Ti Y. F. Guo G. D. Fan G. T. Chen F. R. (2019). Influence of Ceramic Debris on Osteoblast Behaviors: An *In Vivo* Study. Orthop. Surg. 11 (5), 770–776. 10.1111/os.12496 31464084 PMC6819169

[B25] Sun Z. Zeng J. Wang W. Jia X. Wu Q. Yu D. (2020). Magnoflorine Suppresses MAPK and NF-Κb Signaling to Prevent Inflammatory Osteolysis Induced by Titanium Particles *In Vivo* and Osteoclastogenesis via RANKL *In Vitro* . Front. Pharmacol. 11, 389. 10.3389/fphar.2020.00389 32300300 PMC7142243

[B26] Taki N. Tatro J. M. Nalepka J. L. Togawa D. Goldberg V. M. Rimnac C. M. (2005). Polyethylene and Titanium Particles Induce Osteolysis by Similar, Lymphocyte-independent, Mechanisms. J. Orthop. Res. 23 (2), 376–383. 10.1016/j.orthres.2004.08.023 15734251

[B27] Visgauss J. D. Perrin D. L. Wilson D. A. Griffin A. M. Wunder J. S. Ferguson P. C. (2020). Midterm Success of a Custom, Non-fluted, Diaphyseal, Press-Fit Stem Used with a Tumor Megaprosthesis System. J. Arthroplasty 35 (5), 1333–1338. 10.1016/j.arth.2019.12.032 32067897

[B28] Wada T. Nakashima T. Hiroshi N. Penninger J. M. (2006). RANKL-RANK Signaling in Osteoclastogenesis and Bone Disease. Trends Mol. Med. 12 (1), 17–25. 10.1016/j.molmed.2005.11.007 16356770

[B29] Walia B. Lingenheld E. Duong L. Sanjay A. Drissi H. (2018). A Novel Role for Cathepsin K in Periosteal Osteoclast Precursors during Fracture Repair. Ann. N. Y Acad. Sci. 1415 (1), 57–68. 10.1111/nyas.13629 29479711

[B30] Walsh M. C. Lee J. Choi Y. (2015). Tumor Necrosis Factor Receptor- Associated Factor 6 (TRAF6) Regulation of Development, Function, and Homeostasis of the Immune System. Immunol. Rev. 266 (1), 72–92. 10.1111/imr.12302 26085208 PMC4799835

[B31] Wang X. Jiang X. Yu X. Liu H. Tao Y. Jiang G. (2017). Cimifugin Suppresses Allergic Inflammation by Reducing Epithelial Derived Initiative Key Factors via Regulating Tight Junctions. J. Cel Mol Med 21 (11), 2926–2936. 10.1111/jcmm.13204 28597545 PMC5661257

[B32] Wooley P. H. (2014). How Has the Introduction of New Bearing Surfaces Altered the Biological Reactions to Byproducts of Wear and Modularity? Clin. Orthop. Relat. Res. 472 (12), 3699–3708. 10.1007/s11999-014-3725-4 24942963 PMC4397759

[B33] Wu C. Wang W. Tian B. Liu X. Qu X. Zhai Z. (2015). Myricetin Prevents Titanium Particle-Induced Osteolysis *In Vivo* and Inhibits RANKL-Induced Osteoclastogenesis *In Vitro* . Biochem. Pharmacol. 93 (1), 59–71. 10.1016/j.bcp.2014.10.019 25449599

[B34] Wu L. Q. Li Y. Li Y. Y. Xu S. H. Yang Z. Y. Lin Z. (2016). Antinociceptive Effects of Prim-O-Glucosylcimifugin in Inflammatory Nociception via Reducing Spinal COX-2. Biomol. Ther. (Seoul) 24 (4), 418–425. 10.4062/biomolther.2015.168 27257008 PMC4930286

[B35] Yang D. Liu T. Jiang G. Hu X. Zheng T. Li T. (2020). Senkyunolide H Attenuates Osteoclastogenesis and Postmenopausal Osteoporosis by Regulating the NF-Κb, JNK and ERK Signaling Pathways. Biochem. Biophys. Res. Commun. 533 (3), 510–518. 10.1016/j.bbrc.2020.09.054 32977943

[B36] Yao L. Wang S. Wei P. Bao K. Yuan W. Wang X. (2019). Huangqi-Fangfeng Protects against Allergic Airway Remodeling through Inhibiting Epithelial-Mesenchymal Transition Process in Mice via Regulating Epithelial Derived TGF-Β1. Phytomedicine 64, 153076. 10.1016/j.phymed.2019.153076 31473579

[B37] Zhai Z. Qu X. Yan W. Li H. Liu G. Liu X. (2014). Andrographolide Prevents Human Breast Cancer-Induced Osteoclastic Bone Loss via Attenuated RANKL Signaling. Breast Cancer Res. Treat. 144 (1), 33–45. 10.1007/s10549-014-2844-7 24481680

[B38] Zhang L. Haddouti E. M. Welle K. Burger C. Kabir K. Schildberg F. A. (2020). Local Cellular Responses to Metallic and Ceramic Nanoparticles from Orthopedic Joint Arthroplasty Implants. Int. J. Nanomedicine 15, 6705–6720. 10.2147/ijn.S248848 32982228 PMC7494401

[B39] Zhang Q. Tang X. Liu Z. Song X. Peng D. Zhu W. (2018). Hesperetin Prevents Bone Resorption by Inhibiting RANKL-Induced Osteoclastogenesis and Jnk Mediated Irf-3/c-Jun Activation. Front. Pharmacol. 9, 1028. 10.3389/fphar.2018.01028 30254586 PMC6142014

[B40] Zhu W. Yin Z. Zhang Q. Guo S. Shen Y. Liu T. (2019). Proanthocyanidins Inhibit Osteoclast Formation and Function by Inhibiting the NF-Κb and JNK Signaling Pathways during Osteoporosis Treatment. Biochem. Biophys. Res. Commun. 509 (1), 294–300. 10.1016/j.bbrc.2018.12.125 30583865

