# Correction: Cimifugin suppresses NF-κB signaling to prevent osteoclastogenesis and periprosthetic osteolysis

**DOI:** 10.3389/fphar.2025.1678203

**Published:** 2025-12-11

**Authors:** Juan Duan, Xuantao Hu, Tao Li, Gen Wu, Pengcheng Dou, Zhengxiao Ouyang

**Affiliations:** 1 Department of Geriatric Internal Medicine, The Second Xiangya Hospital, Central South University, Changsha, China; 2 Deparment of Orthopedics, The Second Xiangya Hospital, Central South University, Changsha, China

**Keywords:** osteoclast, NF-κB, p38, MAPK, aseptic prosthetic loosening, periprosthetic osteolysis, cimifugin

There was a mistake in Figure 1E (80 μM group) and Figure 2C (80 μM group) as published. Due to an error during figure preparation, the image used for the 80 μM group in Figure 1E (TRAP staining of BMMs) and the 80 μM group in Figure 2C (F-actin immunofluorescence) were incorrect and do not represent the appropriate experimental data. The corrected Figure 1 and Figure 2 appears below.

**FIGURE 1:**
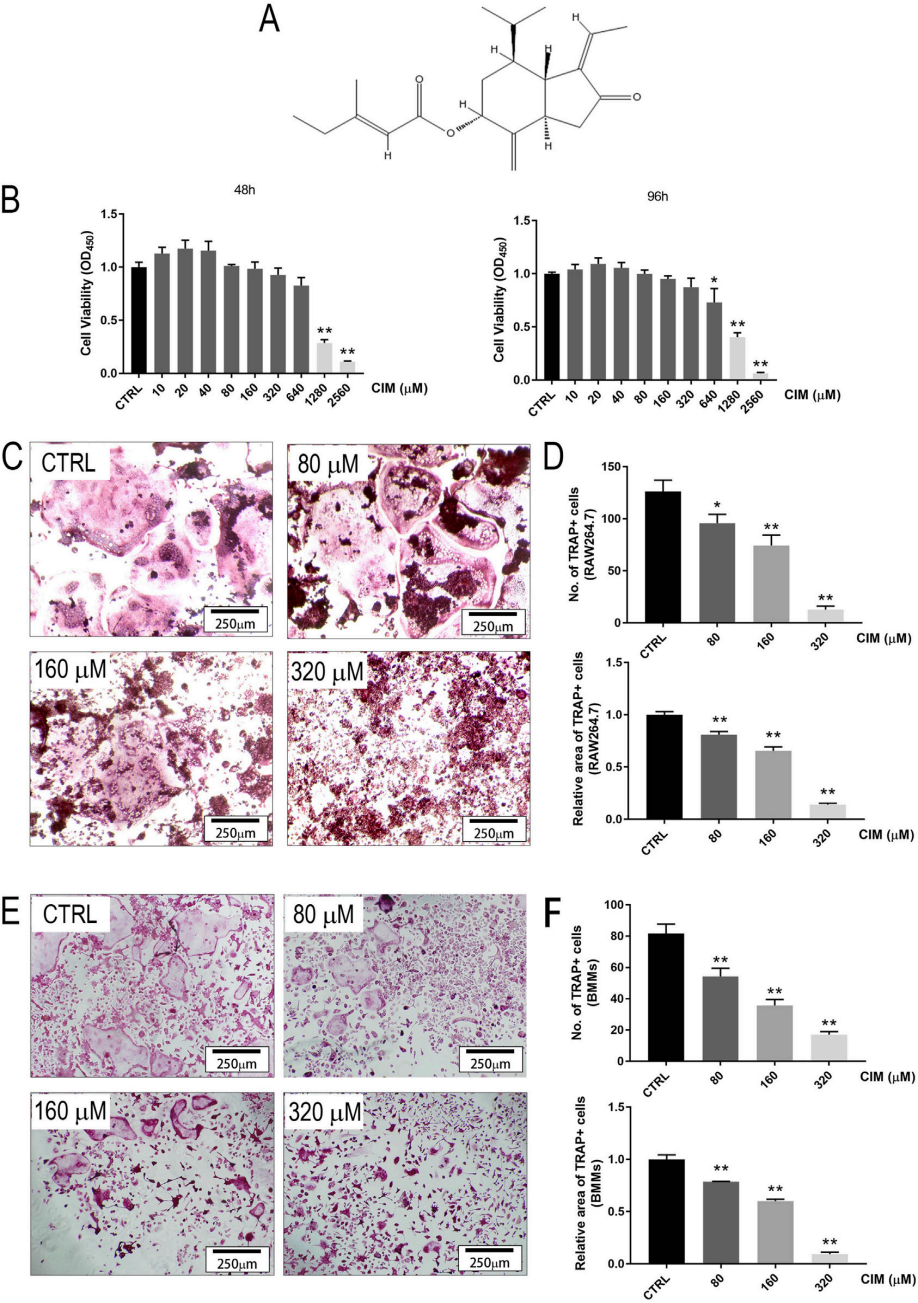
CIM at a noncytotoxic dose inhibits RANKL-induced osteoclast formation in RAW264.7 and BMM cell lineages in a dose-dependent manner. **(A)** The chemical structure of CIM. **(B)** The cell viability of BMMs treated with CIM at different doses for 48 or 96 h. CIM at doses less than or equal to 320 μM was identified as noncytotoxic. **(C)** and **(E)** TRAP staining images of RAW264.7 cells and BMMs incubated with CIM at gradient concentrations for 5–7 days. The numbers **(D)** and areas **(F)** of TRAP + osteoclasts were quantified and analyzed. (*: *p* < 0.05; **: *p* < 0.01 compared with the control group).

**FIGURE 2:**
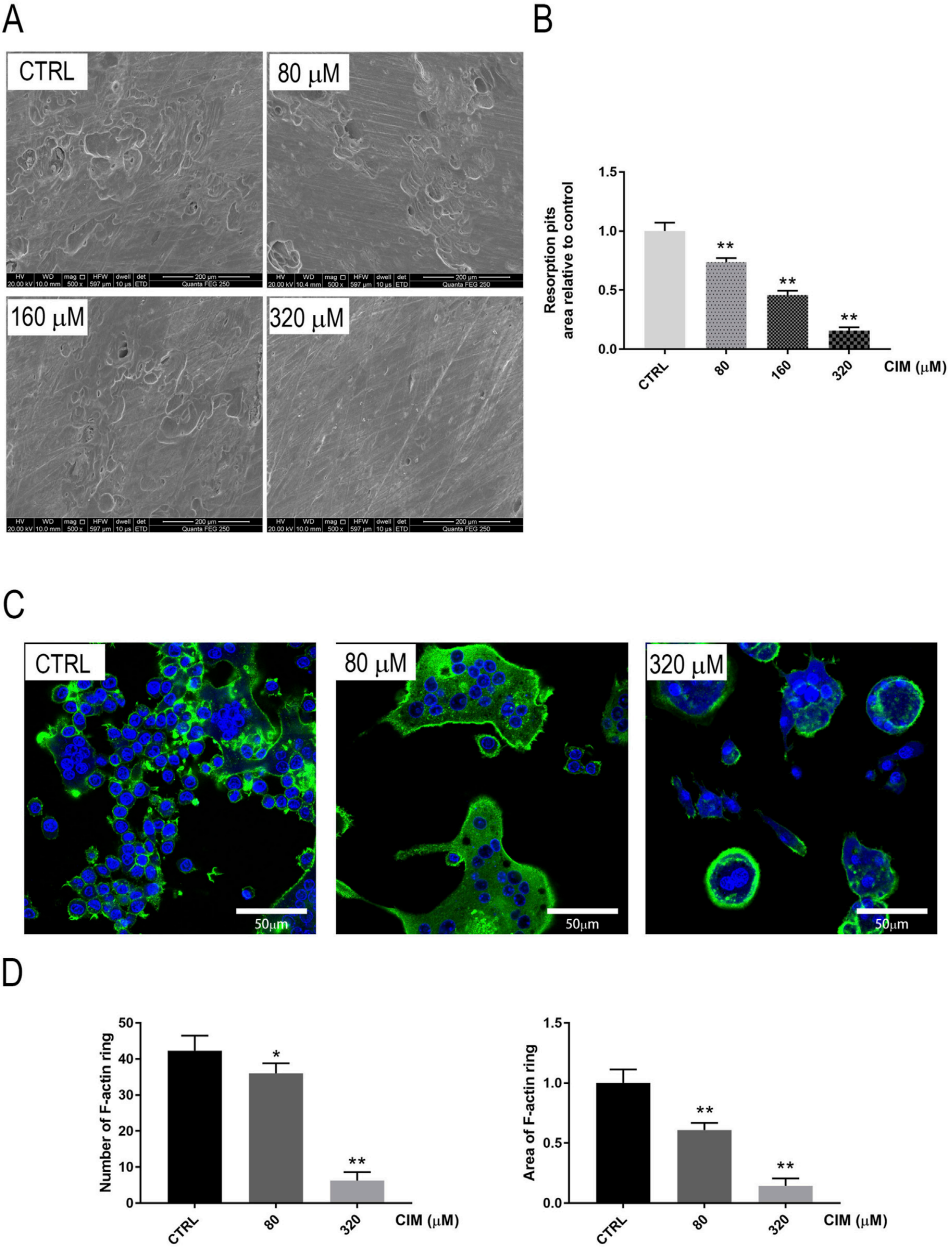
Noncytotoxic CIM dose-dependently mitigates the bone-resorbing activity of osteoclasts in vitro. **(A)** Scanning electron microscopy images of eroded surfaces on bone slices treated with CIM at the indicated concentrations. **(B)** The areas of bone resorption pits relative to the control group are shown. **(C)** Immunofluorescence images of F-actin rings (in green) inBMM-derived osteoclasts treated with CIMat the indicated doses. (**: *p* < 0.01 compared with the control group). **(D)** Number and area of F-actin ring were counted via Image J Software.

The original article has been updated

