# Supplementary figures and images for: Cimifugin Suppresses NF-κB Signaling to Prevent Osteoclastogenesis and Periprosthetic Osteolysis
Source: Front Pharmacol. 2021 Sep 29;12:724256. doi: 10.3389/fphar.2021.724256 (PMC8511420; doi:10.3389/fphar.2021.724256)

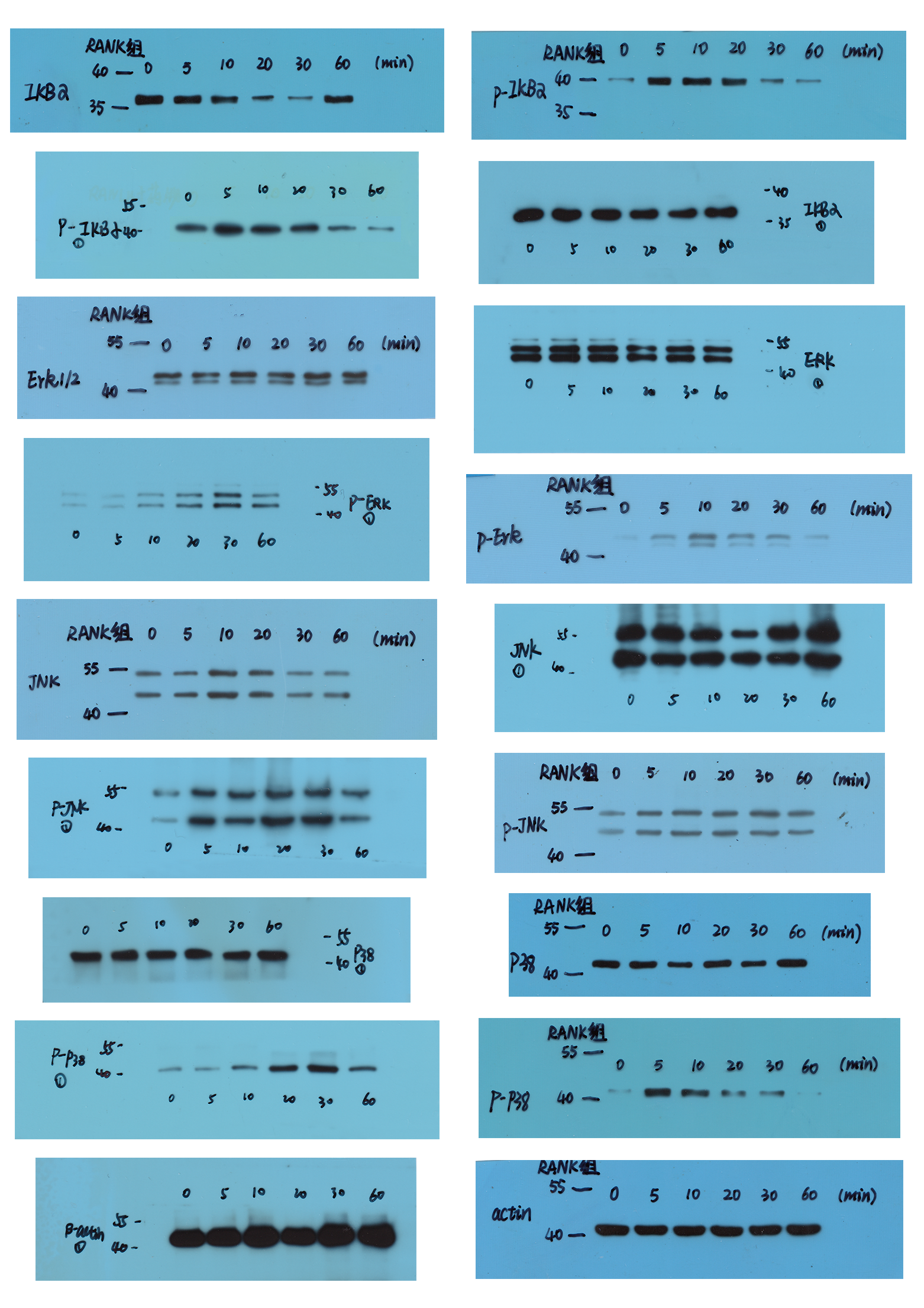

Supplement: Supplementary file 1 [file Image2.TIF]

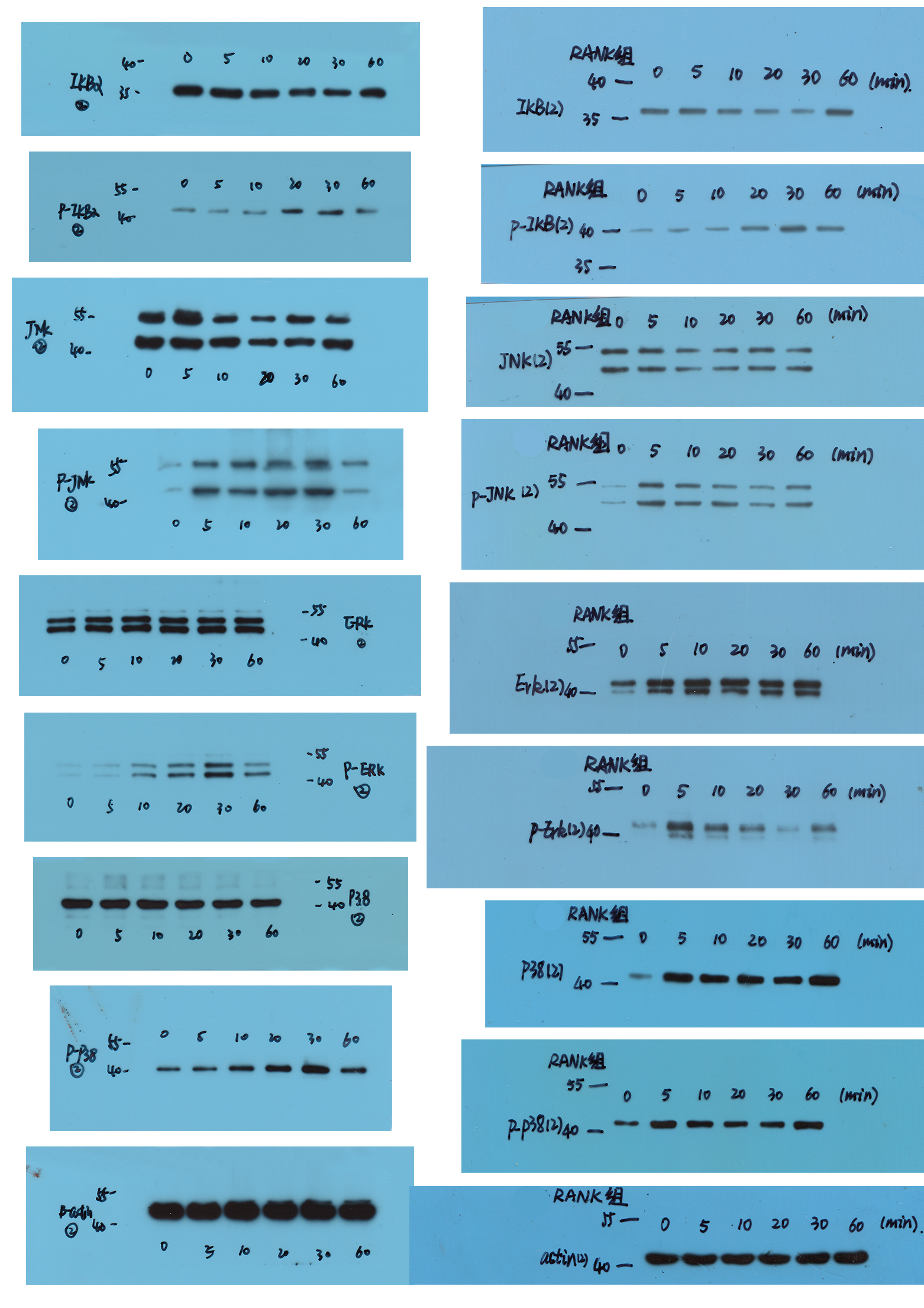

Supplement: Supplementary file 2 [file Image1.TIF]
